# Quality of life changes and their predictors in young adult narcolepsy patients after treatment: A real-world cohort study

**DOI:** 10.3389/fpsyt.2022.956037

**Published:** 2022-08-09

**Authors:** Wei-Chih Chin, Chih-Huan Wang, Yu-Shu Huang, Jen-Fu Hsu, Kuo-Chung Chu, I Tang, Teresa Paiva

**Affiliations:** ^1^Division of Psychiatry and Sleep Center, Chang Gung Memorial Hospital, Taoyuan, Taiwan; ^2^College of Medicine, Chang Gung University, Taoyuan, Taiwan; ^3^Department of Psychology, Zhejiang Normal University, Jinhua, China; ^4^Department of Pediatrics, Chang Gung Memorial Hospital, Taoyuan, Taiwan; ^5^Department of Information Management, National Taipei University of Nursing and Health Sciences, Taipei, Taiwan; ^6^Neurophysiology and Sleep Medicine, University of Lisbon, Lisbon, Portugal

**Keywords:** narcolepsy, daytime sleepiness, quality of life, attention, vigilance

## Abstract

**Background:**

We conducted a five-year prospective follow-up study to track the real-world quality of life of patients with narcolepsy after medication and analyzed predictors.

**Methods:**

The study ultimately included 157 participants who completed 5-year follow-up, 111 had type 1 narcolepsy (NT1) and 46 had type 2 narcolepsy (NT2). Polysomnography, multiple sleep latency test, actigraphy and HLA-typing were conducted. The Short Form 36 Health Survey Questionnaire (SF-36), the Stanford Center for Narcolepsy Sleep Inventory, the Epworth Sleepiness Scale (ESS), the visual analog for hypersomnolence (VAS), and Conners' Continuous Performance Test were used. Descriptive statistics, repeated measures, and hierarchical linear models were applied for analysis.

**Results:**

Most demographic and clinical data did not significantly differ between groups, but the NT1 group had significantly more overweight, more severe narcoleptic symptoms, more positive HLA typing, shorter mean sleep latency, and more sleep onset rapid eye movement periods. No significant change to the physical domains of SF-36 was found in the total group, but we observed significant changes in emotional role functioning and social function. The NT1 group showed significant improvements in physical role functioning, emotional role functioning, and social function. The NT2 group demonstrated significant improvements in emotional role functioning. At the baseline, the NT2 group had significantly better scores, but there was no significant group difference after treatment, except for physical and social function. ESS and VAS were significantly improved during follow-up. At the baseline, the NT1 group had significantly higher ESS and VAS scores, and continuously significantly higher ESS scores during follow-up. Narcolepsy types, HLA typing, age of onset, symptom severity, attention and vigilance were significantly correlated with SF-36.

**Conclusion:**

Symptom control greatly associates with the quality of life in narcoleptic patients, and medication can play the most important role. Management targeting narcoleptic symptoms, attention impairment, and drug adherence should be provided.

## Introduction

Narcolepsy is a chronic sleep-wakefulness disorder characterized by hypersomnolence. Symptoms include cataplexy, hypnogogic/hypnopompic hallucination, sleep paralysis, and disturbed nighttime sleep (1). The 2nd edition of the International Classification of Sleep Disorders (ICSD-2) classified narcoleptic patients into two subtypes by the presence or absence of cataplexy, which refers to sudden and transient muscle weakness usually triggered by emotion (2). Later, based on the absence of hypocretin, a fundamental marker of the most precisely defined category of the disorder, the 3rd edition of the International Classification of Sleep Disorders (ICSD-3) divided narcolepsy into type 1 (NT1) and type 2 (NT2) (3). In addition to abnormal sleep latency and the presence of two or more sleep-onset rapid eye movement periods (SOREMP) by the multiple sleep latency test (MSLT), NT1 has the presence of cataplexy and/or a low or absent cerebrospinal fluid (CSF) hypocretin-1 level. With similar findings in the MSLT, NT2 lacks cataplexy and either the CSF hypocretin-1 level has not been measured or is >110 pg/ml. However, few studies have discussed the significance and impact of this new classification, (4). and its real life impact on narcoleptic patients is not yet clear.

Broughton et al. (5) first studied the influence of narcolepsy on patients' life and noted that their social interaction, work performance, education and achievements, leisure activity, and daily living were poorer than those of normal controls (5). Daniels et al. used the Short-Form-36-Health-Survey-questionnaire (SF-36) to show that these patients had significantly worse scores in physical function, physical role functioning, vitality, general health, body pain, emotional role functioning, psychological health, and social function (6). Although patients with narcolepsy undoubtedly have a poorer quality of life, the quality of life of the newly classified NT1 and NT2 by ICSD-3 has not been fully investigated, nor observed with long-term follow-up. Some studies of the quality of life of NT1 and NT2 revealed incongruent results. A recent study reported that the physical role functioning score of SF-36 was lower in NT1 than in NT2 and controls (7), while another study showed no difference in the total score between NT1 and NT2 (8).

Several factors may influence long-term quality of life, including narcolepsy type, disease duration, symptom severity, and the impact of medication. Previous studies found that patients with a longer disease duration had worse physical role functioning, general health, vitality, social functioning, and emotional role functioning (9) and poorer psychological adjustment and self-esteem if the patients experienced a longer duration between onset and diagnosis (10). However, Ozaki et al. found patients without cataplexy experienced a lower impact on their quality of life than those with cataplexy, as well as had improved mental health the longer the disease duration (11). More severe hypersomnolence could relate to lower physical role functioning, and only the severity of hypersomnolence after treatment could predict general health and vitality in patients with narcolepsy with cataplexy (12). In contrast, Vignatelli et al. found that symptom-related factors were unable to predict the quality of life both before and after treatment (13). Some studies found significant improvement in quality of life after 1 year of pharmacological treatment (14, 15), but surprisingly, two five-year cohort studies showed no significant change before and after the five-year treatment, and some even showed deterioration (12, 13). Overall, these findings are still inconsistent and predictors for the quality of life of patients with narcolepsy were not confirmed.

Neurocognitive function can also play a part, as supported by studies of patients with chronic diseases (16–18). Lower quality of life could relate to more neurocognitive impairment, while improved neurocognitive function led to improvement in quality of life. Narcoleptic patients were found to have significant attention impairment (19), as well as more accidents due to poor vigilance (20). Our previous study found that patients with NT1 had more impairment in attention and vigilance, as well as more severe somnolence, compared with those with NT2 (21, 22). Attention and vigilance may be influenced by somnolence, and can also impact patients' quality of life. At present, studies of neurocognitive impairment and its impact on the quality of life of narcoleptic patients are still lacking.

Questions remain about the quality of life with a newly classified diagnosis of narcolepsy and possible predictors. Most previous studies have been cross-sectional studies, and the diagnosis of narcolepsy was primarily based on ICSD-2 diagnostic criteria (6–9, 23, 24). Longitudinal studies only followed these patients before and after one-year (14, 25, 26) or two-year treatment (27). Two five-year studies only compared these patients' conditions at the beginning of the study and at the five-year follow-up (12, 13). Besides, although the stability of excessive daytime sleepiness in NT1 has been reported (4, 28–32), most studies of the treatment effect of daytime sleepiness of medication such as Modafinil only follow the treatment effect for a short period of time (14, 15, 25, 26). A cohort study reported improvement in daytime sleepiness after treatment by the Epworth Sleepiness Scale (ESS), but only reported the first and fifth year data (12). A more detailed and thorough understanding of the quality of life and symptom control of narcoleptic patients along the course of the disease and the impact of the new ICSD-3 classification may help us to understand patients' difficulties and provide them with timely help.

The quality of life of patients with narcolepsy can vary between NT1 and NT2. It can also fluctuate in the disease course and can be influenced by multiple factors. This study is a five-year prospective cohort study that investigates the changes of the quality of life and the symptom severity of NT1 and NT2 patients. We also analyzed the possible predictors of long-term quality of life.

## Methods

### Participants

We prospectively recruited patients with narcolepsy in the sleep medicine clinic of the Linkou branch of Chang Gung Memorial Hospital from 2013–2019. The diagnosis of narcolepsy was made by experienced sleep medicine doctors according to ICSD-3 diagnostic criteria (3). **Inclusion criteria** consisted of patients (1) newly diagnosed with NT1 or NT2, (2) aged between 16 to 45, (3) that had not received any medication treatment (such as Methylphenidate or Modafinil) and were drug naïve before enrollment, and (4) that were able to cooperate with examinations and complete yearly follow-up questionnaires. **Exclusion criteria** were as follows: (1) PSG showed another severe sleep disorder that may contribute to daytime sleepiness, such as severe obstructive sleep apnea (OSA), (2) neurological disease history, such as epilepsy, stroke, or brain injury, (3) severe cardiovascular disease history, such as hypertension and heart disease, (4) intellectual disability history, and (5) patients with a shift work or circadian rhythm disorder. Informed consent was obtained from all participants, and the study was approved by the institutional review board of Chang Gung Memorial Hospital (CGMH #103-7075A3, 201702299A3C601 and 201902163A3). All subjects and their legal representatives received a detailed explanation of the study and provided their written informed consent prior to entering this study.

**Figure 1:**
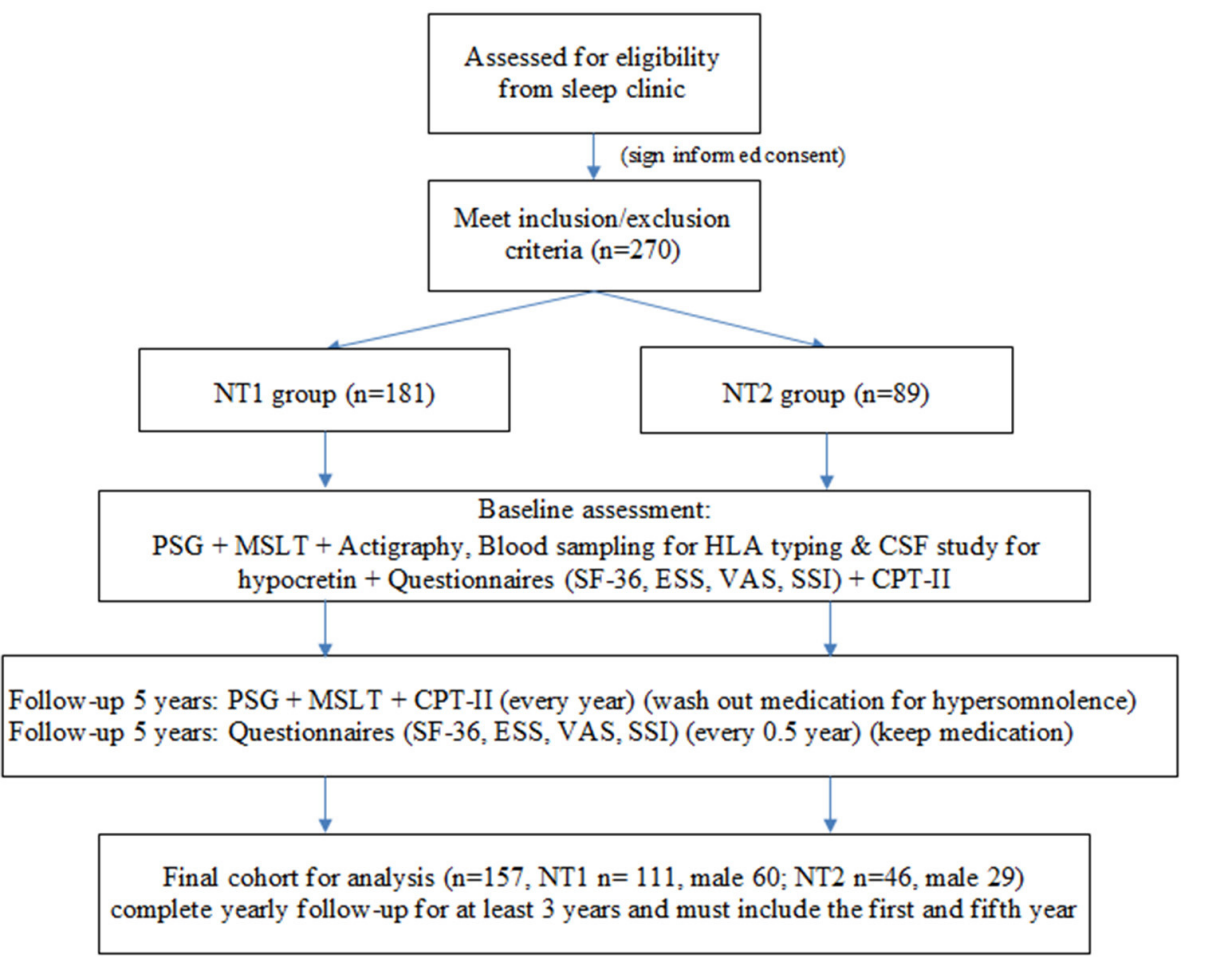
Study flow chart. NT1, type 1 narcolepsy; NT2, type 2 narcolepsy; PSG; polysomnography; MSLT, multiple sleep latency test, CSF; cerebrospinal fluid; SF-36; Short Form 36 Health Survey Questionnaire; ESS; Epworth Sleepiness Scale; VAS; Visual Analog Scale for hypersomnolence; SSI; Stanford Center for Narcolepsy Sleep Inventory; CPT-II; Conners' Continuous Performance Test- II.

A total of 270 patients with narcolepsy who met the inclusion and exclusion criteria were enrolled at the baseline, 181 patients in the NT1 group and 89 in the NT2 group (Figure 1). The PSG, MSLT and actigraphy were performed to confirm the diagnosis of narcolepsy, and repeated yearly due to the requirements for the application of Modafinil under our health insurance system. Blood sampling was done for HLA typing (DQB1 0602), (4). and some received cerebrospinal fluid study for hypocretin. All participants completed questionnaires, including the SF-36 to evaluate quality of life, the Epworth Sleepiness Scale (ESS) and the visual analog scale (VAS) for hypersomnolence, and the Stanford Center for Narcolepsy Sleep Inventory (SSI) (the 5th section) for cataplexy. Our participants also received Conners' Continuous Performance Test- II (CPT-II) to evaluate their attention and vigilance every year. The SF-36, ESS, VAS, and SSI were performed every 6 months during the five-year follow-up. At last, we included 157 participants for the final analysis. All of them completed yearly follow-up for at least 3 years, and the first and fifth year follow-up must be included.

### Medication

Modafinil, 200 mg once daily, or Methylphenidate, 10 mg two to three times per day, were prescribed for hypersomnolence after the baseline work-up and evaluation. Under the current public health insurance system, patients must have regular clinic follow-ups and examinations (PSG and MSLT) to qualify for the annual application of Modafinil. Cataplexy was treated with antidepressants according to doctors' clinical judgment. Patients had to discontinue the medication for hypersomnolence at least 1 week before PSG, MSLT, and CPT-II test. Questionnaires were filled in regular clinic visits to evaluate their real life condition.

### Examinations

#### Polysomnography (PSG)

PSG data was collected on a 32-channel recording system (Embla N7000, Covidien, Ontario, Canada) with continuous video monitoring, according to the criteria of the American Academy of Sleep Medicine (2), including electroencephalography (four leads), electrooculography, electromyography (chin and leg), respiration recorded with nasal cannula/pressure transducer, mouth thermistor, thoracic and abdominal plethysmography bands, neck microphone, diaphragmatic-intercostal muscles electromyography and finger pulse oximetry.

#### Multiple sleep latency test (MSLT)

During the test, participants try to fall asleep every 2 h in a total of five trials (10:00, 12:00, 14:00, 16:00 and 18:00). Collected variables include sleep latency and sleep onset REM periods (SOREMPs). In patients with narcolepsy, the MSLT shows a mean sleep latency of 8 min or less, and two or more SOREMPs (33).

#### Conners' continuous performance test- II (CPT-II):

CPT-II was used to investigate the cognitive performance of participants aged six or older with inattention, poor vigilance, impulsivity, and hyperactivity (34). It can help to determine whether conditions improve or deteriorate with medication. It takes <15 min to administer and has few practice effects (35). Higher scores suggest poorer attention, vigilance, or impulse control. We have published the CPT-II results of patients with narcolepsy in previous studies (21, 22).

#### HLA typing (HLA DQB1^*^0602):

All participants received the HLA DQB1^*^0602 haplotype blood test, which has been associated with narcolepsy. HLA is a gene region encoding the major histocompatibility complex protein located in chromosome six and divided into three sub-regions (class I, II, and III). Studies by Mignot and colleagues have shown that narcolepsy with cataplexy is highly associated with HLA DQA1^*^01:02 and HLA DQB1^*^0602 in all ethnic populations (36).

#### Questionnaires

- The Short Form 36 Health Survey questionnaire (SF-36) evaluates eight domains: (1) physical functioning, evaluating physical restriction by the disease, (2) physical role functioning, evaluating the occupational role restriction by the disease, (3) body pain, evaluating the severity of pain, (4) general health, evaluating subjective health condition, (5) vitality, evaluating subjective vitality, (6) social functioning, evaluating social function restrictions by the disease, (7) emotional role functioning, evaluating occupational role restriction due to emotional problems, and (8) mental health, evaluating subjective mental health condition. Higher scores suggest a better quality of life and less restriction by the disease.
- The Epworth Sleepiness Scale (ESS) was developed by Johns (37) to evaluate the severity of daytime sleepiness (37). We used it and the visual analog scale (VAS) to evaluate daytime sleepiness and changes during follow-ups. Higher scores suggest more severe daytime sleepiness.
- The Stanford Center for Narcolepsy Sleep Inventory (SSI) was developed by the Stanford Sleep Center in 1993, (38). with nine different sections and a total of 146 items. We used the fifth section of the SSI to evaluate the severity of cataplexy (39).

### Statistical analysis

We used SPSS 19.0 (IBM, 2011) and HLM 6.20 (SSI, 2010) to analyze our data. Demographic data is presented as number, mean, percentage, and standard deviation. We used the chi-square test for group comparisons of percentages and independent *t-*test for the mean. A *p*-value of <0.05 was considered statistically significant. We used hierarchical linear modeling (HLM) to analyze the continuous change of SF-36 and its correlation with different variables, as well as of individual differences. Doing so also helped us to manage missing values. We analyzed fixed effects in level one and random effects in level two. In level one, we set the quality of life as the dependent variable and symptom severity as the independent variable. In level two, we included sex, age at inclusion, age of onset, disease duration, and HLA typing as independent variables. The intraclass correlation was the proportion of individual variance from the total variance.

## Results

Table 1A shows the demographic data of the NT1 and NT2 groups, which has been published before (4). Of the 157 participants, 57% were male, the average age of onset was 13.5 ± 5.6 years, and the current age was 23.9 ± 8.7 years. Thirty-four percent of participants were obese, and body mass index (BMI) was 22.9 ± 6.2. Of all participants, 67 had positive HLA typing. The MSLT results showed mean sleep latency to be 3.1 + 2.3 min and SOREMP to be 3.8 + 1.1 times. Table 1B shows the comparison of the baseline data of the included participants of final cohort analysis and the drop-out participants. Only emotional role functioning (*p* = 0.03) and parasomnia symptom (*p* = 0.005) are significantly different.

**Table 1A:** Demographic and clinical data of narcolepsy type 1, narcolepsy type 2, and total participants.

	**Total (*n* = 157)**	**Type 1(*n* = 111)**	**Type 2(*n* = 46)**	***p*-value**
Gender	Male 89 (57%)	Male 60 (54%)	Male 29 (63%)	0.301
	Female 51(43%)	Female 51 (46%)	Female 17 (37%)	
Current age	23.90 ± 8.73	23.89 ± 8.74	23.94 ± 8.73	0.458
Age of onset (years)	13.45 ± 5.57	13.15 ± 5.29	14.16 ± 6.14	0.340
BMI (at entry)	22.87 ± 6.21	23.45 ± 6.61	21.48 ± 5.26	0.084
Overweight (at entry) (BMI≥25)	Yes: 46 (34%)	Yes: 46 (41%)	Yes: 7 (15%)	0.002^*^
Hypersomnolence	Yes:157 (100%)	Yes:111 (100%)	Yes: 46 (100%)	-
Cataplexy	Yes: 111 (70%)	Yes: 111 (100%)	Yes: 0 (0%)	<0.001^*^
Hypnogogic hallucination	Yes:103 (66%)	Yes: 85 (77%)	Yes: 18 (39%)	<0.001^*^
Sleep paralysis	Yes: 105(67%)	Yes :84 (76%)	Yes :21 (46%)	<0.001^*^
Parasomnia	Yes:102 (65%)	Yes: 80 (72%)	Yes: 22 (48%)	0.004^*^
REM behavior symptoms	Yes: 8(5%)	Yes: 6 (5%)	Yes: 2 (4%)	0.593
Disturbed night sleep	Yes: 42 (27%)	Yes: 31 (28%)	Yes: 11 (24%)	0.605
HLA_DQB1_0602	Yes: 105 (67%)	Yes: 91 (82%)	Yes: 14 (30%)	<0.001^*^
MSLT: mean sleep latency (min)	3.13 + 2.32	2.37 ± 1.85	4.95 ± 2.36	<0.001^*^
MSLT: number of SOREMPs (times)	3.80 + 1.10	4.04 ± 1.00	3.24 ± 1.14	<0.001^*^

Student t-test of type 1 and type 2 narcolepsy, ^*^p-value <0.05.

BMI, body mass index; REM, rapid eye movement; HLA, human leukocyte antigen; MSLT, multiple sleep latency test; SOREMP, number of sleep-onset rapid-eye-movement period.

**Table 1B:** Demographic and clinical data of included and narcolepsy type 1, narcolepsy type 2, and total participants.

	**Included participants (*n* = 157)**	**Drop-out participants (*n* = 113)**	***p*-value**
Gender	Male 89 (56.69%)	Male 57 (50.44%)	0.310
Current age	23.26 ± 8.64	24.10 ± 9.00	0.438
Age of onset (years)	13.45 ± 5.14	14.89 ± 6.80	0.113
BMI (at entry)	23.32 ± 4.99	23.80 ± 5.19	0.487
Obesity (at entry) (BMI≥25)	53 (33.8%)	31 (27.4%)	0.268
Hypersomnolence	157 (100%)	113 (100%)	-
Cataplexy	111 (70.7%)	67 (59.29%)	0.063
Hypnogogic hallucination	103 (65.61%)	60 (53.1%)	0.060
Sleep paralysis	105 (66.88%)	67 (59.29%)	0.201
Parasomnia	102 (64.97%)	58 (51.33%)	0.030^*^
REM behavior symptoms	8 (5.1%)	13 (11.5%)	0.052
Disturbed night sleep	42 (26.75%)	33 (29.2%)	0.657
HLA_DQB1_0602	105 (66.88%)	65 (57.52%)	0.116
MSLT: mean sleep latency (min)	3.56 ± 3.06	3.75 ± 3.28	0.638
MSLT: SOREMs (times)	3.72 ± 1.20	3.44 ± 1.49	0.118
SF-36 PF	75.57 ± 30.91	74.87 ± 34.28	0.860
SF-36 RP	26.59 ± 36.22	35.18 ± 39.62	0.070
SF-36 BP	73.18 ± 30.1	72.81 ± 35.35	0.925
SF-36 GH	45.96 ± 26.4	44.38 ± 26.87	0.632
SF-36 VT	36.66 ± 22.53	33.27 ± 22	0.220
SF-36 PH	50.57 ± 22.54	46.12 ± 26.3	0.147
SF-36 RE	26.11 ± 39.46	41 ± 43.87	0.005^*^
SF-36 SF	50.16 ± 28.45	51.33 ± 33.92	0.766
ESS	16.24 ± 4.01	16.1 ± 3.89	0.772
VAS	82.53 ± 14.9	79.12 ± 12.88	0.095

Student t-test of included and drop-out participants, ^*^p value <0.05.

BMI, body mass index; REM, rapid eye movement; HLA, human leukocyte antigen; MSLT, multiple sleep latency test; SOREMP, number of sleep-onset rapid-eye-movement period.

SF-36, The Short Form 36 Health Survey Questionnaire; PF, physical functioning; RP, role functioning-physical; BP, body pain, GH: general health; VT, vitality; PH, psychological health; RE, role functioning-emotional; SF, social functioning; ESS, Epworth sleepiness scale; VAS, visual analog scale.

Among the 157 participants, 111 had NT1, and 46 had NT2; 57% of them were male. The NT1 group had more overweight (*p* = 0.002), cataplexy (*p* <0.001), hypnogogic hallucinations (*p* <0.001), sleep paralysis (*p* < 0.001), and parasomnia (*p* = 0.004). The results of HLA typing revealed more positive cases in the NT1 groups (*p* < 0.001), and MSLT showed a shorter mean sleep latency (*p* < 0.001) and more SOREMPs (*p* < 0.001).

**Figure 2:**
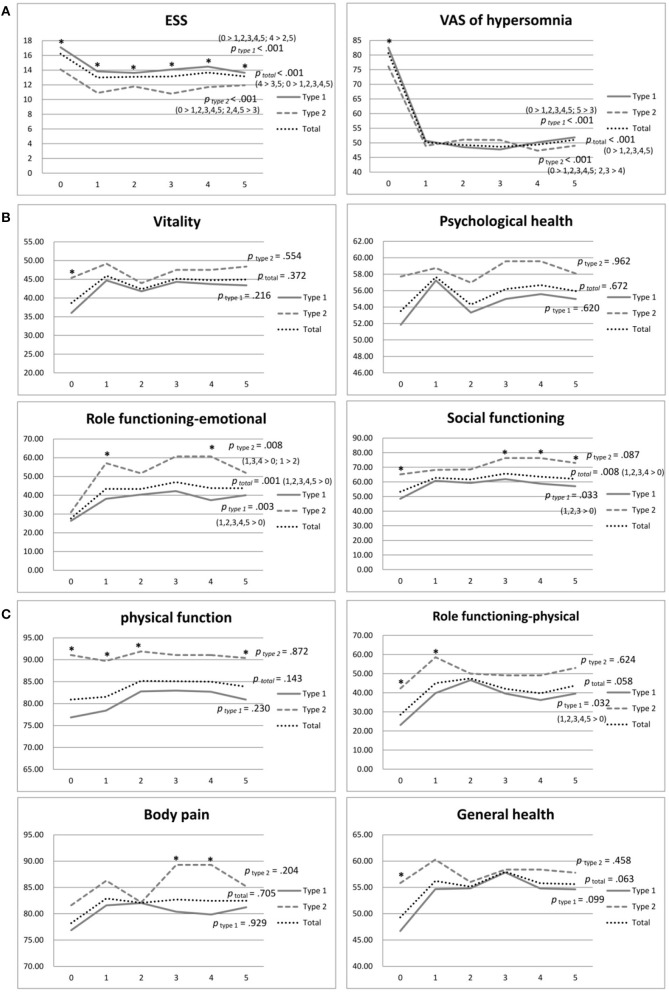
**(A)** Physical domains of SF-36 of narcolepsy type 1, narcolepsy type 2, and total participants during the 5-year follow-up. ^*^p <0.05. **(B)** Psychological domains of SF-36 of narcolepsy type 1, narcolepsy type 2, and total participants during the 5-year follow-up. ^*^p value <0.05. **(C)** ESS and VAS of narcolepsy type 1, narcolepsy type 2, and total participants during the 5-year follow-up. ^*^p value <0.05. ESS, Epworth Sleepiness Scale; VAS, Visual Analog Scale for hypersomnolence.

Table 2 shows the data of SF-36 (physical and psychological domains), ESS, and VAS of total narcolepsy, NT1, and NT2 (Figures 2A–C). We observed no significant changes in the physical domains of SF-36 in the total group, although physical role functioning and general health demonstrated improvements (Figure 2A). With regard to psychological domains, significant improvements were found in emotional role functioning (*p* = 0.001) and social functioning (*p* = 0.008) (Figure 2B). Furthermore, ESS (*p* < 0.001) and VAS significantly improved in the total group (*p* < 0.001) (Figure 2C).

**Table 2:** Comparison of the data of SF-36, ESS and VAS of narcolepsy type 1 and type 2.

	**0 y**			**1 y**			**2 y**			**3 y**			**4 y**			**5 y**			**ANOVA**	
**SF-36**	**Type 1**	**Type 2**	***p* ^1^**	**Type 1**	**Type 2**	***p* ^1^**	**Type 1**	**Type 2**	***p* ^1^**	**Type 1**	**Type 2**	***p* ^1^**	**Type 1**	**Type 2**	***p* ^1^**	**Type 1**	**Type 2**	***p* ^1^**	**Type 1 *p*^2^**	**Type 2 *p*^2^**
PF	76.86 ± 25.20	91.07 ± 16.69	**<0.0**01^*^	78.41 ± 24.73	89.71 ± 16.18	0.003^*^	82.78 ± 20.74	91.90 ± 14.42	0.012^*^	82.97 ± 22.48	91.07 ± 18.63	0.091	82.70 ± 23.47	91.07 ± 18.63	0.093	80.91 ± 24.61	90.40 ± 13.69	0.030^*^	0.230	0.872
RP	23.10 ± 33.20	42.26 ± 42.59	0.011^*^	39.84 ± 38.73	58.57 ± 43.70	0.021^*^	46.60 ± 44.33	50.00 ± 41.19	0.719	39.56 ± 37.47	49.11 ± 44.35	0.272	36.15 ± 38.41	49.11 ± 44.35	0.148	39.55 ± 41.30	53.00 ± 42.89	0.186	0.032* (1,2,3,4,5 >0)	0.624
BP	76.88 ± 24.72	81.61 ± 21.20	0.278	81.59 ± 21.64	86.29 ± 21.11	0.275	82.04 ± 21.78	82.16 ± 24.61	0.981	80.38 ± 19.78	89.29 ± 15.23	0.017^*^	79.86 ± 24.65	89.29 ± 15.23	0.023^*^	81.23 ± 22.74	85.20 ± 19.09	0.450	0.929	0.204
GH	46.71 ± 24.55	55.83 ± 21.69	0.037^*^	54.67 ± 24.28	60.29 ± 22.42	0.237	54.81 ± 25.41	56.03 ± 20.80	0.817	57.85 ± 24.00	58.39 ± 20.86	0.915	54.80 ± 23.86	58.39 ± 20.86	0.484	54.64 ± 26.19	57.80 ± 23.37	0.606	0.099	0.458
VT	36.00 ± 18.88	45.36 ± 22.51	0.011^*^	44.67 ± 18.65	49.14 ± 22.34	0.257	41.79 ± 22.01	43.97 ± 20.85	0.644	44.30 ± 21.31	47.50 ± 19.60	0.488	43.72 ± 19.60	47.5 ± 19.6	0.386	43.36 ± 19.98	48.40 ± 20.55	0.303	0.216	0.554
PH	51.85 ± 18.46	57.71 ± 17.13	0.078	57.23 ± 19.61	58.74 ± 17.05	0.689	53.33 ± 21.68	56.97 ± 17.09	0.366	54.99 ± 20.15	59.57 ± 13.52	0.184	55.57 ± 20.13	59.57 ± 13.52	0.334	54.98 ± 19.85	58.08 ± 14.01	0.484	0.620	0.962
RE	26.35 ± 39.95	30.95 ± 39.23	0.527	38.10 ± 43.48	57.14 ± 43.96	0.030^*^	40.33 ± 44.94	51.72 ± 44.17	0.242	42.19 ± 42.94	60.71 ± 41.63	0.051	37.39 ± 42.00	60.71 ± 41.63	0.014^*^	40.00 ± 45.54	52.00 ± 40.92	0.263	0.003* (1,2,3,4,5 >0)	0.008* (1,3,4 > 0; 1 > 2)
SF	48.45 ± 24.31	65.18 ± 24.93	<0.001^*^	60.71 ± 26.39	68.21 ± 27.34	0.160	59.26 ± 25.98	68.53 ± 29.43	0.114	61.87 ± 25.39	76.34 ± 23.16	0.009^*^	58.78 ± 23.57	76.34 ± 23.16	0.001^*^	57.05 ± 26.87	73.00 ± 25.18	0.014^*^	0.033* (1,2,3 > 0)	0.087
ESS	17.08 ± 3.33	14.19 ± 4.76	<0.001^*^	13.82 ± 4.50	11.05 ± 4.16	<0.001^*^	13.61 ± 3.81	11.81 ± 3.32	0.006^*^	14.07 ± 3.67	10.80 ± 3.60	<0.001^*^	14.46 ± 3.31	11.69 ± 3.05	<0.001^*^	13.63 ± 2.54	11.94 ± 3.22	0.001^*^	<0.001* (0 > 1,2,3,4,5; 4 > 2,5)	<0.001* (0 > 1,2,3,4,5; 2,4,5 > 3)
VAS	82.51 ± 16.15	76.11 ± 16.36	0.026^*^	50.72 ± 21.59	48.97 ± 18.43	0.608	48.53 ± 20.10	51.07 ± 15.61	0.397	47.74 ± 16.81	50.95 ± 12.76	0.195	50.18 ± 18.68	47.35 ± 11.41	0.249	51.88 ± 15.15	49.00 ± 10.67	0.179	<0.001* (0 > 1,2,3,4,5; 5 > 3)	<0.001* (0 > 1,2,3,4,5; 2,3 > 4)

P^1^, Independent sample t test; P^2^, Repeated measure ANOVA; ^*^p value <0.05.

PF, physical functioning; RP, physical role functioning; BP, body pain; GH, general health; VT, vitality; PH, psychological health; RE, emotional role functioning; SF, social functioning; SF-36, Short Form 36 Health Survey Questionnaire; ESS, Epworth Sleepiness Scale; VAS, Visual Analog Scale for hypersomnolence.

Higher scores of domains of SF-36 suggest better quality of life. Higer scores of ESS and VAS suggest more severe daytime sleepiness.

The NT1 group showed significant improvements in physical role functioning ( *p*= 0.032), emotional role functioning (*p* = 0.003), and social functioning (*p* = 0.033) during the five-year follow-up period (Table 2; Figures 2A,B). The NT2 group showed significant improvement in emotional role functioning ( *p* = 0.008) (Table 2; Figure 2B). At the baseline, the NT2 group had a significantly better score in most domains than the NT1 group (Table 2; Figures 2A,B). During follow-up, although the NT2 group had better scores, the differences between groups decreased after medication. At the fifth year of follow-up, no significant difference in physical domains was found between the two groups, except physical function (*p* = 0.03), and we observed no significant difference in psychological domains except social function (*p* = 0.014) (Table 2, Figures 2A,B).

Both the NT1 and NT2 groups showed significant improvement in ESS and VAS during the five-year follow-up (both *p* < 0.001) (Table 2; Figure 2C). At the baseline, the NT1 group had significantly higher scores in both ESS (*p* < 0.001) and VAS (*p*= 0.026) than the NT2 group (Table 2; Figure 2C). During the follow-up period, the NT1 group continuously had significantly higher scores than the NT2 group in ESS, but not in VAS (Table 2; Figure 2C).

In Table 3, we analyzed the factors associated with the SF-36 of patients with narcolepsy as possible predictors using the full hierarchical linear model. Gender, current age, and disease duration were not significantly correlated with any domains of the SF-36. The different narcolepsy types (NT2) were correlated with the physical health summary scale, physical function, physical role functioning, general health, psychological health, and vitality. Positive HLA typing (DQB1 0602) was correlated with the physical health summary scale, physical role functioning, mental health summary scale, social function, vitality, and emotional role functioning. Age of onset was correlated with the mental health summary scale, psychological health, and vitality.

**Table 3:** Predictors of SF-36 of patients with narcolepsy using the full hierarchical linear model.

	**Fixed effect**	**PHS**	**PF**	**RP**	**BP**	**GH**	**MHS**	**PH**	**SF**	**VT**	**RE**
A	**Gender**	−3.704	−4.940	−6.719	−3.335	−0.853	−4.527	−4.884	−8.690	−5.342	0.955
	**Type1/2**	10.540^*^	13.172^*^	14.571^*^	4.859	13.752^*^	6.783	11.095^*^	5.427	15.283^*^	3.626
	**Current age**	0.163	−0.024	−0.477	−0.485	−0.283	−0.275	−0.254	−0.064	−0.364	−0.437
	**Age of onset**	0.627	−0.649	−0.423	−0.763	−0.727	−0.613^*^	−0.772^*^	−0.220	−1.130^*^	−0.463
	**Disease duration**	−0.075	−0.021	−0.505	−0.343	−0.056	−0.103	0.091	0.016	0.027	−0.424
	**HLA typing (DQB1 0602)**	−9.316^*^	−7.667	−10.619^*^	−9.263	−8.354	−13.931^***^	−5.664	−11.392^*^	−11.319^*^	−28.338^***^
	**CPT:Attention**	0.110	0.057	0.239	0.061	0.111	0.144^*^	0.063	0.169^*^	0.027	0.270^*^
	**CPT:Vigilance**	0.122	0.050	0.284^*^	0.117	0.251^*^	0.299^*^	0.104	0.391^*^	0.089	0.609^***^
	**CPT:Impulsivisity**	0.112	0.098	0.210	0.015	0.120	0.083	0.079	0.075	0.047	0.096
B	**Time**	−0.115	−0.266	−0.891	0.363	0.835	1.506^*^	0.165	0.418	0.383	4.4.7^*^
	**Cataplexy**	−0.585^**^	−0.621^***^	−1.551^**^	−0.335	−0.333	−0.495^*^	−0.338^*^	−1.001^*^	−0.627^**^	−0.557
	**ESS**	−0.783^**^	−0.703^***^	−0.952^*^	−0.279	−1.077^*^	−1.963^***^	−0.922^**^	−1.157^***^	−0.979^**^	−1.692^*^
C	**Variance components**	133.761	285.268	424.278	193.013	256.764	321.387	422.908	337.490	316.902	385.981
	***χ2***	277.381	532.572	167.429	298.327	373.352	150.192	158.640	263.223	140.680	140.772
	***p***	<0.001	<0.001	<0.001	<0.001	<0.001	<0.001	<0.001	<0.001	<0.001	<0.001

A, Between individual level; B, Within individual level; C, Random-Effects Model.

PHS, physical health summary score; PF, physical functioning; RP, physical role functioning; BP, body pain; GH, general health; MHS, mental health summary scale; VT, vitality; PH, psychological health; RE, emotional role functioning; SF, social functioning; CPT, Conners' Continuous Performance Test- II.

Conditional growth model.

Level 1, *qualityoflife = β_00_+β_10_(TIME)+β_20_(ESS)+β_30_(Cata)+ γ_00_*.

Level 2, *β_i_ = γ_00_+γ_i0_(Between−individuallevelvariance)+ μ_i_*.

* *p* value < .05;

** *p* value < .01;

*** *p* value < .001.

Furthermore, the attention and vigilance domains were correlated with the mental health summary scale, social function, and emotional role functioning (Table 3). The vigilance domain was also correlated with physical role functioning, general health, mental health summary scale, social function, and emotional role functioning. The impulsivity domain did not show any significant correlation.

ESS was correlated with all domains of SF-36, except body pain (Table 3). Cataplexy (the fifth section of the SSI) was correlated with most domains, except body pain, general health, and emotional role functioning (Table 3).

## Discussion

This five-year prospective cohort study aimed to track the quality of life of young adult narcolepsy patients after treatment. Our previous study presented annual objective MSLT and PSG results for narcolepsy patients for up to 5 years (4) but these objective findings could not fully reveal the real-world life of patients with narcolepsy. They did not represent patients' subjective feelings such as quality of life. In this study, we evaluated patients' subjective condition and the differences in the quality of life and symptom severity between NT1 and NT2 by questionnaires. We used a hierarchical linear model to analyze the possible predictors of quality of life with five-year collected data, including subjective questionnaires and attention tests. Objective findings by PSG and MSLT were not always consistent with subjective findings. Abnormal objective findings were persistently present during our yearly follow-ups, especially in those with type 1 narcolepsy, but subjective quality of life could fluctuate after treatment.

Our results showed that the physical domains did not significantly change during follow-up in patients with narcolepsy (Figure 2A), consistent with the results of Vignatellli et al. (13), indicating that narcolepsy cannot be cured by medication alone. The scores of physical role functioning and general health were relatively lower than other domains at the baseline. Despite improvement trends, only physical role functioning was improved in the NT1 group (Table 2; Figure 2A). Therefore, an exercise program can be developed and recommended for patients with narcolepsy. OSA and obesity are commonly comorbid with narcolepsy, (40) and treatment of narcolepsy should also address these physical conditions to prevent further exacerbation of physical health.

Among the psychological domains of SF-36, the emotional role functioning and social functioning of patients with narcolepsy significantly improved after treatment during the five-year follow-up (Table 2; Figure 2B), but vitality and psychological health were not changed despite improved daytime sleepiness (Figure 2B). Dodel et al. found that the stigma of narcolepsy could lead to more of a psychological influence in these patients (23). Psychiatric comorbidities such as depression and anxiety were not uncommon (22, 41) and can lead to poor psychological health and fatigue. Besides medication for daytime sleepiness and cataplexy, mental health care and intervention, such as antidepressants for depression and anxiety, counseling, or group therapy, can be helpful and should be provided if needed.

At the baseline, the NT2 group had better scores in all domains of SF-36 than the NT1 group, although the difference was not always significant (Table 2; Figures 2A,B). The NT2 group also had significantly less daytime sleepiness than the NT1 group (Table 2; Figure 2C). Both NT1 and NT2 belong to the spectrum of central hypersomnia, and the symptom severity of NT1 is more severe than NT2. Our study revealed that the NT2 group also had a better quality of life than the NT1 group. These subjective results were consistent with the objective findings of our previous studies of PSG, MSLT, and brain imaging (4, 21, 22). However, among the domains of SF-36, the NT2 group scored relatively lower in physical role functioning, general health, vitality, psychological health, and emotional role functioning (Table 2), with only significant improvement in emotional role functioning during follow-up. Therefore, though better than the NT1 group, the NT2 group did not have the same degree of response to the current treatment and further investigation of confounding factors is needed.

Furthermore, although the NT1 group had lower scores in most domains of SF-36, the group differences of SF-36 decreased after medication during the five-year follow-up. In the fifth year of follow-up, only physical function and social function were significantly lower in the NT1 group. The quality of life of the NT1 group may respond better to medication and related decreased narcoleptic symptoms than the NT2 group. These findings highlight the fundamental role of medication in the treatment of narcolepsy and show that benefits are also found in the improvement in quality of life, not just narcoleptic symptom control.

The severity of daytime sleepiness evaluated using both ESS and VAS showed improvement during the five-year follow-up after treatment (Table 2; Figure 2C). Most previous studies have also shown improved daytime sleepiness after treatment but have only compared the results between the baseline and 1 year after treatment (25, 26, 42). However, Ozaki et al. compared the results of the baseline and the fifth year of follow-up and found that daytime sleepiness had increased after the five-year treatment in those with narcolepsy with cataplexy but observed no change in daytime sleepiness in those with narcolepsy without cataplexy (12). We also found some fluctuation in daytime sleepiness in the total group and both the NT1 and NT2 groups (Figure 2C). Possible causes include medication tolerance and under-estimation in subjective measurements. It may also be related to compliance and dosage. Such medication as Modafinil and Methylphenidate plays an important role in the treatment of daytime sleepiness, but some patients may need a higher dosage as they grow or develop tolerance (43), an issue that requires further study. Pharmaco-education should be implemented to enhance drug adherence.

Since many factors can be associated with long-term quality of life, we used the full hierarchical lineal model to analyze possible predictors. We found most physical and psychological domains of SF-36 were correlated with the severity of daytime sleepiness and cataplexy (Table 3), consistent with previous studies (9, 11–13, 15, 23). In general, with less daytime sleepiness and cataplexy, the quality of life of narcoleptic patients is better, and vice versa. Besides the predictable physical influence of cataplexy, daytime sleepiness also influenced most physical domains, except body pain. Previous studies also showed daytime sleepiness had a predictable effect on physical role functioning and general health, but not body pain (9, 11–13). These findings further emphasize the importance of regular medication to control narcoleptic symptoms as much as possible.

We found that attention and vigilance were correlated with some domains of SF-36 of narcoleptic patients (Table 3), but not impulsivity. Better attention and vigilance bring better quality of life. Studies by Findley et al. and Fronczek et al. both found that the major neurocognitive impairment in narcoleptic patients is vigilance, not attention (20, 44). Our results indicated that vigilance had a broader impact on quality of life than attention or impulse control. In addition to daytime sleepiness and cataplexy, future studies of the development of new medication for narcolepsy can consider including improvement in attention and vigilance among the outcome measures.

Similar to previous studies of narcolepsy with and without cataplexy, we found that the different types of narcolepsy were correlated with domains of SF-36, including physical function, physical role functioning, general health, psychological health, and vitality (Table 3). Patients with NT2 could be predicted to have a better quality of life in these domains. Our results also showed that positive HLA typing (HLA DQB1^*^06:02) had a significant negative correlation with several domains of SF-36, including physical role functioning, social function, vitality, and emotional role functioning (Table 3). Therefore, NT1 and positive HLA typing could predict poorer quality of life, consistent with our previous findings of brain imaging (21, 22). NT1 has been proved to be a well-defined entity, but NT2 presented clear clinical and test variability, especially in MSLT, and most NT1 had positive HLA typing (4). Our results suggest that different types of narcolepsy and HLA typing can help clinicians predict the prognosis in patients with narcolepsy, and HLA typing should be included in routine narcolepsy work-ups.

Among the demographic variables, different from the previous study (9), disease duration was not significantly correlated with any domains of SF-36. Only age of onset was correlated with psychological health and vitality. Surprisingly, patients with an earlier age of onset can have a better quality of life in terms of psychological health and vitality, as demonstrated by the relatively young patient population of our study. In this adolescent and young adult population, development issues are of concern, and narcolepsy may have a different impact at different development stages. Sleepiness in childhood can be less disturbing than in adolescence, since peer pressure and academic stress all increase in adolescence. Better family support can also play a role in the early detection and improved understanding of the disease. This is an interesting area worth further exploration.

Declining quality of life can lead to the termination of treatment, depression and anxiety, low achievement, and even suicide (40, 41, 45). Therefore, monitoring the quality of life of patients with narcolepsy should be included in clinical practice. Treatment of narcolepsy cannot depend on medication alone. Non-pharmacological management targeting narcoleptic symptoms and attention impairment should be developed and provided. Planned short naps and sleep hygiene can help patients to decrease daytime sleepiness. Neurocognitive training can be helpful for those with neurocognitive impairment, such as patients with ADHD (46, 47), but further study is warranted for patients with narcolepsy. Psychoeducation can help patients understand the correlation of symptom severity and quality of life, as well as improve therapeutic rapport, increase medication adherence, and relieve anxiety and depression.

This study has some limitations. First, we only used data of subjective measurements and analyzed their correlation with SF-36. Scoring sleepiness and cataplexy done by patients could present individual differences. Second, changes in symptom severity and quality of life could be disturbed by poor compliance, and thus we excluded a number of participants with irregular follow-up for the final analysis. However, although patients who were included for the analysis had regular clinic follow-ups and assessments, we still could not confirm medication adherence. Third, under the current healthcare system, Mondafinil 200 mg is the maximum dosage we can prescribe for all patients, and said dosage may be inadequate for some. Besides, medications for narcolepsy were prescribed according to patients' needs. Our patients were relatively young and many were still under development. Their medications can change during follow-up. Other medications for narcolepsy such as Sodium Oxybate or Pitolisant are currently not available in Taiwan. Fourth, many possible confounding factors may influence quality of life, and some are not included in our analysis. For examples, comorbidities are common for narcoleptic patients, and related medication treatments can also have impact on the daytime sleepiness. To decrease possible negative impacts, comorbidities such as depression and anxiety were treated and managed as well as possible. Besides, excluding patients with comorbidities may result in incomplete presentation of the real life of narcoleptic patients. Last, it can be very difficult to follow healthy controls for long periods of time, and thus we did not recruit a control group for the five-year follow-up. Despite its limitations, this cohort study reflects the real-world condition of young adult patients with narcolepsy and can provide practical information for clinicians and medical professionals.

## Conclusion

There is still a long way to go for young adult patients with narcolepsy, and our results confirm that symptom control greatly associates with the ir quality of life. Medication can play the most important role. Managements targeting narcoleptic symptoms and attention impairment, as well as psychosocial intervention to increase rapport and drug adherence, should be provided to improve their quality of life.

## Data availability statement

The raw data supporting the conclusions of this article are available from the corresponding author upon reasonable request.

## Ethics statement

This study was approved by the Institutional Review Board of Chang Gung Memorial Hospital (CGMH #103-7075A3, 201702299A3C601, and 201902163A3). All subjects and their legal representatives received a detailed explanation of the study and provided their written informed consent prior to entering this study.

## Author contributions

Conception and design of the work: Y-SH and TP. Data collection: Y-SH, C-HW, and W-CC. Data analysis: C-HW, K-CC, and IT. Article draft: W-CC and Y-SH. Manuscript editing and critical revision of the article: W-CC, Y-SH, and J-FH. Final approval of the version to be published: Y-SH. All authors contributed to the article and approved the submitted version.

## Funding

This study was partially supported by Chang Gung Memorial Hospital Research Grants (CMRPG3J0132) awarded to Y-SH and W-CC and the Taiwan Ministry of Science and Technology Grant #: MOST 109-2314-B-182A-112-MY3 awarded to Y-SH.

## Conflict of interest

The authors declare that the research was conducted in the absence of any commercial or financial relationships that could be construed as a potential conflict of interest.

## Publisher's note

All claims expressed in this article are solely those of the authors and do not necessarily represent those of their affiliated organizations, or those of the publisher, the editors and the reviewers. Any product that may be evaluated in this article, or claim that may be made by its manufacturer, is not guaranteed or endorsed by the publisher.
